# Modulating food intake by nasal application of peptides targeting melanocortin 4 receptor and ghrelin receptor systems

**DOI:** 10.1093/braincomms/fcaf450

**Published:** 2026-01-21

**Authors:** Benginur Özbay, Eva-Maria Jülke, Moritz List, Marcin Nowicki, Sylvia Els-Heindl, Kerstin Immig, Karin Mörl, Ingo Bechmann, Annette G Beck-Sickinger

**Affiliations:** Faculty of Medicine, Institute of Anatomy, University of Leipzig, Leipzig 04103, Germany; Faculty of Life Sciences, Institute of Biochemistry, University of Leipzig, Leipzig 04103, Germany; Faculty of Life Sciences, Institute of Biochemistry, University of Leipzig, Leipzig 04103, Germany; Faculty of Medicine, Institute of Anatomy, University of Leipzig, Leipzig 04103, Germany; Faculty of Life Sciences, Institute of Biochemistry, University of Leipzig, Leipzig 04103, Germany; Faculty of Medicine, Institute of Anatomy, University of Leipzig, Leipzig 04103, Germany; Faculty of Life Sciences, Institute of Biochemistry, University of Leipzig, Leipzig 04103, Germany; Faculty of Medicine, Institute of Anatomy, University of Leipzig, Leipzig 04103, Germany; Faculty of Life Sciences, Institute of Biochemistry, University of Leipzig, Leipzig 04103, Germany

**Keywords:** intranasal peptide delivery, MC4R agonist, ghrelin receptor modulation, hypothalamic targeting, appetite regulation

## Abstract

The regulation of appetite by pharmaceuticals has gained significant interest for the treatment of obesity and cachexia. The melanocortin 4 receptor (MC4R) and the ghrelin receptor (GhrR) are known to play a crucial role in the regulation of energy homeostasis. Thus, peptide ligands, which modulate these receptors, have become attractive therapeutic lead structures. A key challenge is the efficient delivery of such peptides to the targeted receptors, which are expressed in the hypothalamus. Therefore, direct nose-to-brain delivery is a compelling strategy. Here, we report on food intake that is modulated by using intranasal applied peptides. We synthesized fluorescently labelled variants of the MC4R agonist setmelanotide, the GhrR agonist ghrelin (Ghr) and the GhrR inverse agonist KbFwLL-NH_2_ [β-(3-benzothienyl)-D-alanine (b)] and assessed their receptor activity. Further, we measured the permeability and stability of these peptides on Calu-3 cells as a model system for the nasal mucosa. Next, the uptake of peptides after intranasal application was analysed *in vivo* by quantification of fluorescent signals in the olfactory bulb, cortex and hypothalamus. In addition, we monitored the effects of the two most promising peptides on food intake *in vivo*. Although no significant changes in body weight were observed, we detected differences in the daily change in food intake: this parameter was reduced for mice treated with setmelanotide variants and increased for mice treated with GhrR agonists compared to a control group. Taken together, our findings clearly underline the high potential of intranasal peptide administration for modulating food intake.

## Teaser

Özbay *et al.* demonstrated that intranasal administration of peptides targeting melanocortin 4 and ghrelin receptors can modulate food intake in mice. Their study shows that selected peptides successfully reach the hypothalamus, supporting the potential of nasal peptide delivery for treating obesity and cachexia.

## Graphical Abstract

**Graphical Abstract:**
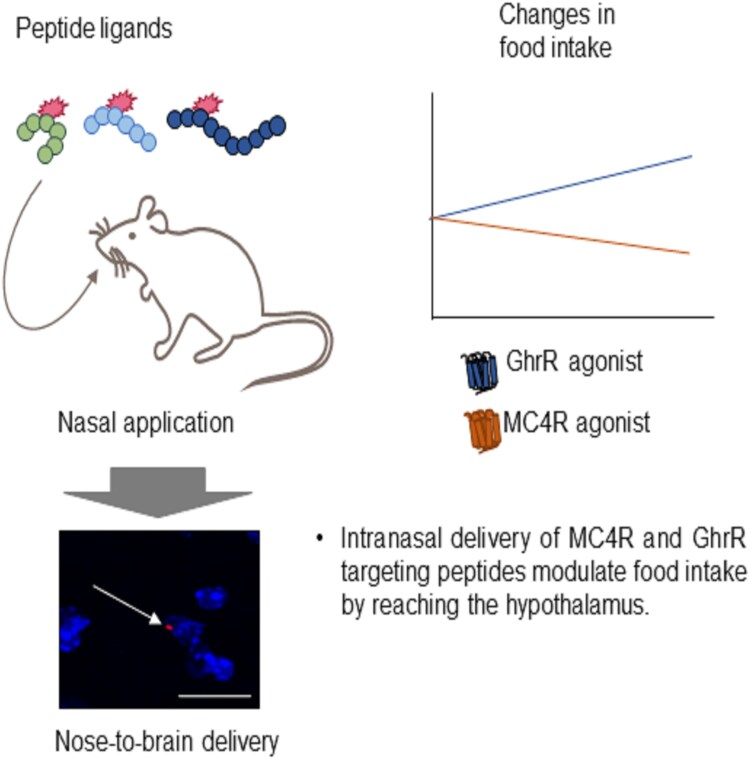

## Introduction

Obesity is a widespread and growing global health concern that significantly contributes to chronic diseases such as diabetes or cardiovascular pathologies, associated with high morbidity and mortality. According to World Health Organization (WHO), 2.5 billion adults aged 18 years and older have been characterized to be overweight in 2022, including over 890 million adults who were living with obesity.^1^ Obesity not only reduces quality of life but also imposes considerable economic burdens on healthcare systems.^2^ Conversely, conditions like cachexia and anorexia, characterized by severe weight loss and malnutrition, present a further set of health challenges, highlighting the complex nature of food intake and energy homeostasis.^3^ Despite broad public health initiatives and research efforts, the rates of obesity and other metabolic diseases continue to rise, pointing out the need for innovative and effective interventions.

**Figure 1:**
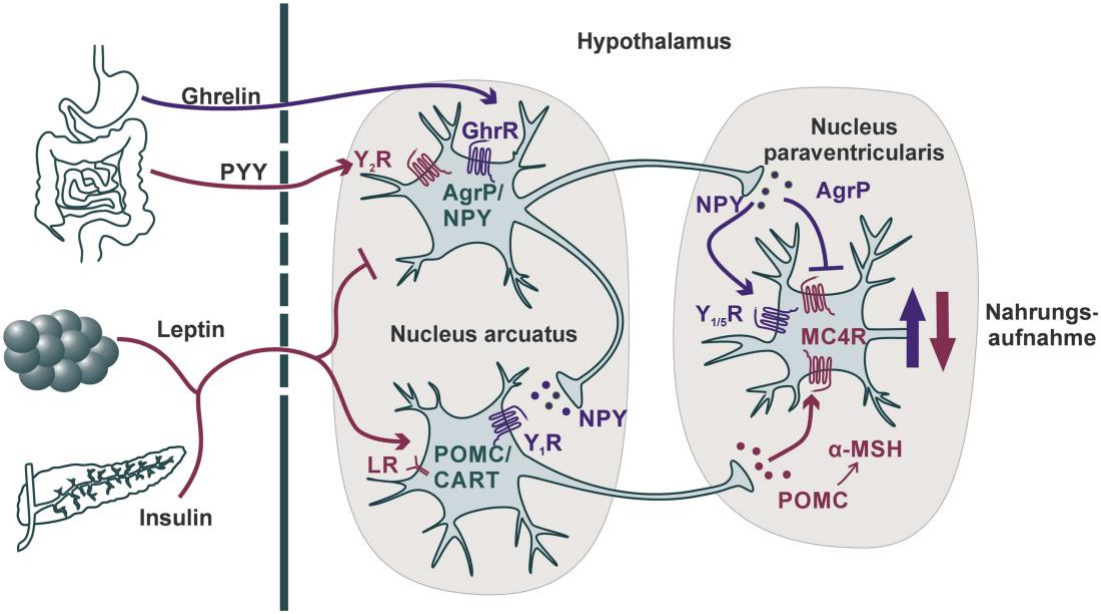
**Regulation of food intake within the hypothalamus.** Peptide hormones from the gastrointestinal tract (Ghr, PYY), fat tissue (leptin) and the pancreas (insulin) modulate activity of the neurons located within the accurate nucleus. Those further transmit signals by segregation of neuropeptides to neurons within the PVN nucleus. Peptides and receptors promoting food intake are marked in purple, and those reducing food intake are displayed in red. AgRP, agouti-related peptide; CART, cocaine- and amphetamine-regulated transcript; GhrR, ghrelin receptor; LR, leptin receptor; MC4R, melanocortin 4 receptor; α-MSH, α-melanocyte-stimulating hormone; NPY, neuropeptide Y; POMC, proopiomelanocortin; PYY, peptide YY; Y_1/2/5_R, neuropeptide Y receptor 1/2/5.

The hypothalamus regulates the energy homeostasis. Particularly, the arcuate (ARC) and the paraventricular (PVN) nuclei are responsible for the appetite regulation (Fig. 1).^4,5^ Activation of the melanocortin 4 receptor (MC4R), expressed on neurons within the PVN, suppresses food intake.^6^ The agouti-related peptide (AgRP) is an antagonist of the MC4R.^7^ In contrast, the α-melanocyte-stimulating hormone (α-MSH), enzymatically processed from proopiomelanocortin (POMC),^8^ activates the MC4R. This signalling pathways are countered by neuropeptide Y (NPY), which can interact with the G_i_-coupled neuropeptide Y receptors 1 and 5 (Y_1_R and Y_5_R) of PVN neurons.^9,10^ The release of NPY and AgRP from neurons of the ARC (ARC^AgRP^) is stimulated by growth hormone secretagogue receptor 1α (GHSR1α), also called ghrelin receptor (GhrR) after its native ligand ghrelin (Ghr).^10^ Ghr is released from the stomach while fasting; however, the GhrR itself also promotes strong constitutive activity leading to the stimulation of appetite.^11-16^ In contrast, the neuropeptide Y receptor 2 (Y_2_R), activated by peptide YY (PYY) from the gastrointestinal tract, reduces ARC^AgRP^ neuron activity und thereby causes an anorectic effect.^17^ Further, leptin from adipose tissue, insulin released from pancreas and their respective receptors can influence activity of ARC^AgRP^ neurons as well as POMC/cocaine- and amphetamine-regulated transcript (CART) (ARC^POMC^) neurons.^18,19^ Those ARC^POMC^ neurons reduce food intake, as released POMC induces activation of MC4R in the PVN. Further, feedback loops between ARC^POMC^ and ARC^AgRP^ neurons fine-tune those signals. For example, NPY from ARC^AgRP^ neurons activates Y_1_R expressed on ARC^POMC^ neurons, downregulating their activity.^4^

Peptides can be used to modulate the activity of the receptors involved in appetite control. This includes the orexigenic effect of Ghr. However, the constitutive activity of the GhrR can be reduced by the inverse agonist KbFwLL-NH2 [β-(3-benzothienyl)-D-alanine (b)] to repress food intake. A similar biological effect is promoted by the MC4R agonist setmelanotide.^20^

In general, peptides are emerging as promising candidates for drug development, especially due to combining high activity, selectivity and low immunogenity.^21^ Yet their therapeutic application encounters significant challenges such as limited oral bioavailability, high metabolic activity and inability of crossing the blood–brain barrier (BBB).^22,23^ Delivering drugs directly to the hypothalamus by the nasal route offers a promising therapeutic approach.^24^ This method has gained considerable interest due to its capacity to bypass the BBB.^23,25^ Moreover, intranasal administration allows for direct delivery to the brain by avoiding liver metabolism, renal filtration, gastrointestinal degradation and serum breakdown.^26^ In addition, intranasal delivery minimizes systemic exposure and associated side effects, providing a safer alternative to conventional administration, and the simplicity of this delivery method also improves patient engagement.^27^ Several studies have already demonstrated the suitability of intranasal delivery of neuropeptides in both clinical and preclinical contexts. For instance, intranasal insulin has been shown to reduce snack intake and increase satiety in women^28^ and improve memory function in both young and elderly adults.^29^ Similarly, intranasal administration of oxytocin at 24 IU has been shown to reduce food intake in rodents without affecting locomotor activity or blood glucose levels, supporting its selective central effects on appetite regulation.^30^ Moreover, neuromedin-U receptor agonists applied intranasally suppressed weight gain in mice at doses of 100–250 µg/day.^31^

The aim of this study is to detect peptides involved in the regulation of energy homeostasis in the hypothalamus following intranasal administration. Therefore, peptides targeting the MC4R and the GhrR have been fluorescently labelled and characterized regarding receptor activity, permeability on a model system for the nasal mucosa and stability. Promising candidates were applied intranasally and tracked *in vivo* using fluorescent imaging. Further, we investigate the effects of these peptides on food intake in mice through daily administration over 14 days, to evaluate their potential for treatment of obesity or cachexia.

## Materials and methods

### Peptide synthesis

Peptides were synthesized using solid-phase peptide synthesis (SPPS) with a fluorenylmethoxycarbonyl (Fmoc)/*tert-*butyl (*t-*Bu) strategy and orthogonal protection groups as described previously.^32-35^ Synthesis protocols are described in the Supplementary material.

Purification of peptides to ≥95% purity was achieved by reversed-phase high-performance liquid chromatography (RP-HPLC) on Nexera (Shimadzu, Kyōto, Japan) equipped with an Aeris Peptide 5 µm XB-C18 column (250 × 21.2 mm, Phenomenex, Torrance, USA). Amount of eluent B [acetonitrile (ACN) (VWR, Radnor, USA) + 0.08% trifluoroacetic acid (TFA) (Sigma-Aldrich, St. Louis, MO, USA)] in eluent A (H_2_O + 0.1% TFA) was gradually increased by 1%/min with a flow rate of 15 mL/min. Analytical RP-HPLC was carried out with an Aeris Peptide 3.6 µm XB-C18 100 Å (250 × 4.6 mm, Phenomenex), a Kinetex 5 μm Biphenyl 100 Å LC column (250 × 4.6 mm, Phenomenex) and a Jupiter Proteo 4 µm 90 Å LC column (250 × 4.6 mm, Phenomenex) on a Hitachi Chromaster (VWR) at 40°C. Eluent B in A was increased by 1.25%/min with a flow rate of 1.55, 1.55 and 0.6 mL for Aeris, Biphenyl and Proteo columns, respectively.

For mass spectrometry, an electrospray ionization (ESI)-Orbitrap system (Thermo Fisher Scientific, Waltham, MA, USA) or matrix-assisted laser desorption ionization time-of-flight (MALDI-TOF, MALDI-TOF-TOF Ultraflex III or Microflex, Bruker, Billerica, USA) was used.

### Cyclic adenosine monophosphate reporter gene assay

HEK293 cells were cultured under standard conditions.^33^ Cyclic adenosine monophosphate (cAMP) reporter gene assay was carried out with HEK293 cells transiently transfected with pGL4.29[luc2P/CRE/Hygro] (Promega, Madison, WI, USA) and pcDNA3_MC4R plasmids. For transfection, a master mix consisting of 0.6 µL Lipofectamine (Invitrogen by Thermo Fisher) per 25 µL Opti-MEM (Gibco, Grand Island, NY, USA) per well was preincubated at room temperature for 5 min and then gently mixed with 0.125 µg of both plasmids per 25 µL Opti-MEM per well and incubated for 30 min at room temperature. Detached HEK293 cells were diluted to 100 000 cells per 150 μL Dulbecco’s modified Eagle’s medium (DMEM, Biowest, Nuaillé, France) mixed 1:1 (*v/v*) with Ham’s F12 (F-12, Biowest) supplemented with 15% heat-inactivated foetal bovine serum (FBS, Biochrom, Berlin, Germany) per well and mixed with the Lipofectamine/plasmid solution. Two hundred microlitres of the mixture was added per well to a white 96-well plate (Greiner Bio-One International GmbH, Kremsmünster, Austria), previously coated with 0.1 mg/mL poly-D-lysine (Sigma-Aldrich) in Dulbecco’s phosphate-buffered saline (DPBS) for 1 h at room temperature. Cells were incubated overnight at humidified atmosphere (37°C, 5% CO_2_). Subsequently, the medium was replaced by 50 μL of fresh DMEM/F12 (1:1) + 15% FBS per well, and cells were incubated for 2.5 h at humidified atmosphere. The medium was removed, and cells were starved in 50 μL of DMEM/F12 (1:1) without FBS at humidified atmosphere for 1 h. One millimolar peptide stocks in dimethyl sulfoxide (DMSO, Sigma-Aldrich) were diluted in DMEM/F12 (1:1) to desired concentrations. Cell medium was discarded, and 50 μL of the peptide dilutions were added. Cells were stimulated for 3 h, washed with DMEM/F12 (1:1) and then incubated for 10 min in 30 μL DMEM/F12 (1:1) per well at room temperature. Thirty microlitres of ONE-Glo luciferase assay substrate, dissolved in ONE-Glo luciferase buffer (Promega) according to manufacturer’s protocol, was added to each well. The plate was transferred to a plate reader (Infinite M200, Tecan Trading AG, Männedorf, Switzerland), briefly shaken and incubated 5 min at room temperature. Measurement of luminescence was carried out for 1000 ms, and luminescence values were normalized to maximal setmelanotide response in GraphPad Prism (Version 10, GraphPad Software, Boston, MA, USA). Non-linear regression was used to calculate half maximal effect (EC_50_) and maximal efficiency (*E*_max_) values.

### Inositol monophosphate accumulation assay

Inositol monophosphate (IP_1_) accumulation assay was carried out as reported previously.^36^ COS-7_hGHSR1α were seeded with 8 000 cells in 20 µL DMEM supplemented with 10% FBS and 400 μg/mL hygromycin B (InvivoGen, Toulouse, France) per well in a white 364-well plate (Greiner) and cultured under humidified atmosphere overnight. Dilution series of peptides from 1 mM DMSO stocks and of IP_1_ cryptate were prepared in Hanks’ balanced salt solution (HBSS, Merck) substituted with 20 mM lithium chloride (Sigma-Aldrich). The medium was discarded and replaced by 15 µL of the peptide solution, while 15 µL of the diluted standard was transferred to empty wells. After incubation at a humidified atmosphere for 1 h for the agonists or 3 h for the inverse agonists, 3 µL of each of the manufacturer’s antibody cryptate and the IP_1_ coupled to the d2 acceptor were added per well. The plate was incubated for 1 h at room temperature, before homogeneous time-resolved fluorescence (HTRF) signals were measured with a plate reader (Spark, Tecan) and calculated by dividing the fluorescent signal of the Förster resonance energy transfer (FRET) acceptor [excitation wavelength (*λ*_ex_) = 320 nm ± 25 nm; emission wavelength (*λ*_em_) = 665 nm ± 8 nm; lag time 100 µs] by the signal of the FRET donor (λ_ex_ = 320 nm ± 25 nm; *λ*_em_ = 620 nm ± 10 nm; lag time 100 µs). Data analysis was carried out with GraphPad Prism. HTRF signals from peptides were normalized to the control peptide, and EC_50_ and *E*_max_ for agonists and minimal efficiency (*E*_min_) for inverse agonists were calculated using non-linear regression curve fit.

### Calu-3 permeability assay and peptide stability

Apparent permeability (*P*_app_) was measured on Calu-3 cells differentiated in translucent 12-well ThinCert inserts with a pore size of 0.4 µm (Greiner Bio-One). Culture and differentiation of Calu-3 cells were reported previously.^33^ Both apical and basolateral compartments were washed twice with assay buffer, which consists of DMEM/F12, phenol red-free (Gibco) supplemented with 1% bovine serum albumin (BSA, Carl Roth), and cells were adjusted in this buffer for 30 min under humidified atmosphere. Peptides in DMSO stocks, fluorescein sodium (Fl, Carl Roth) and fluorescein isothiocyanate (FITC)-dextran [average (av.) mass 4000 Da, FD4, Sigma-Aldrich] were diluted to 31.6 µM in assay buffer. Cells were stimulated apically with 400 µL compound solution, and buffer in the basolateral compartment was replaced with 1.2 mL fresh assay buffer. 0, 6 and 24 h after stimulation, 300 µL samples were taken from the basolateral chamber and directly replaced with fresh buffer. At 24 h, an additional sample was taken from the apical compartment. All samples were transferred in a black 96-well plate (BRAND GmbH, Wertheim, Germany) together with a dilution series of the tested compounds, and 6-carboxytetramethylrhodamine (Tam)-fluorescence (*λ*_ex_ = 542 nm ± 20 nm; *λ*_em_ = 587 nm ± 20 nm) or fluorescein/FITC fluorescence (*λ*_ex_ = 492 nm ± 10 nm; *λ*_em_ = 517 nm ± 10 nm) was measured with a Tecan Spark. Data were copied to GraphPad Prism for linear regression of concentration-fluorescent signals, and apparent permeability was calculated according to the following equation:


$$P_{\mathrm{app}}=\;\frac{\frac{\Delta n}{\Delta t}}{A\;\cdot c_{\mathrm{apical}}}$$

where *P*_app_ is the apparent permeability (cm/s), $\frac{\Delta n}{\Delta t}$ is the flux (mol/s), *A* is the area (cm^2^), and *c*_apical_ is the concentration of apical solution (mol/cm^3^).

Directly before and after the permeability assay, transepithelial electrical resistance (TEER) was measured with an EVOM2 equipped with a STX3 electrode (World Precision Instruments, LLC, Hertfordshire, UK). Data were excluded in case of significantly reduced TEER. One hundred microlitres of the 24 h samples from the apical chamber and reference sample from the diluted peptide stock were mixed with 200 µL ACN/ethanol (1:1, *v/v*; PanReac AppliChem, part of ITW, Chicago, IL, USA) and incubated for at least 12 h at −20°C. After 5 min centrifugation at 12 000*×g*, the supernatant was filtered in a Spin-X Tube (Costar Spin-X Centrifuge Tube, 0.22 μm, Corning, Corning, NY, USA) by centrifugation for 1 h at 12 000*×g*. The sample was diluted 1:1 with H_2_O and analysed using analytical RP-HPLC on a VariTide RPC column (250 mm × 4.6 mm, 200 Å, 6 μm, Agilent Technologies, Santa Clara, CA, USA). Eluent B in eluent A was gradually increased by 1.25%/min with a flow rate of 1 mL/min, and eluents were detected by fluorescence (*λ*_ex_ = 525 nm; *λ*_em_ = 572 nm). The amount of intact peptide was calculated from a relative area under the curve integrated with OpenLab EZChrom (Agilent).

### Animals

Healthy, male C57BL/6N mice from 8 weeks age were housed in groups of two to five per cage in accordance with 2010/63/EU and Society of Laboratory Animal Science (GV SOLAS) guidelines in a climate-controlled room (21°C ± 2°C, 55% ± 15% humidity,12 h light–dark cycles). All experimental procedures were evaluated and accepted by the animal experimental committee and approved by the local authorities (TVV57/20). To reduce confounders, treatments were administered at the same time daily with randomized measurement order. The animals were placed individually in cages during the experiment with free access to food and water. Researchers conducting the experiment were aware of the group assignments. They were monitored daily for general health and predefined exclusion criteria were applied: mice experiencing more than 20% weight loss, signs of severe distress or abnormal mobility removed. These criteria were established to ensure ethical treatment and reliable data collection. However, no animals met the exclusion criteria. The sample size was determined based on previously published data.^37,38^

### Intranasal application

Peptides were administered at concentrations corresponding to 10 times their *in vitro* EC₅₀ values, dissolved in DPBS (Gibco). For each administration, mice were briefly anaesthetized with 2.5% isoflurane (Baxter, Deerfield, IL) and positioned supine at ∼45° angle to optimize intranasal absorption and reduce loss due to gravity. A total volume of 20 μL per mouse was administered (10 μL per nostril) using a calibrated Eppendorf micropipette (Merck KGaA, Darmstadt, Germany), applying the solution dropwise onto the external nasal openings for passive inhalation. Without physical insertion, this non-invasive method minimized the risk of mucosal trauma. Animals were euthanized following completion of the 14-day daily administration protocol.

### Isolation of mouse brains and preparation of the tissues

Following the treatment and observation, the animals underwent euthanasia. Therefore, a Xylazin/Ketamin Cocktail (0.25 mg xylazine, Elanco GmbH, Cuxhaven, Germany, and 2.5 mg ketamine, bela-pharm GmbH, Vechta, Germany, per 25 g mouse) was injected intraperitoneally. The chest cavity was opened for a cardiac puncture, and mice were transcardially perfused with DPBS until the intravascular compartment was cleared from blood. Brains were taken and fixed in 4% paraformaldehyde (PFA, Carl Roth) in DPBS for 24 h. After, 10, 20 and 30% sucrose solutions (Carl Roth) were employed for 24 h each. The brain tissues were sectioned sagittally at 20 µm using a cryostat (Leica Microsystems, Wetzlar, Germany).

### Immunohistochemistry staining

Tissue sections were blocked in 10% (*m/v*) normal goat serum (NGS, Jackson Immunoresearch Europe Ltd., Ely, UK) and 0.3% (*v/v*) Triton (Roche Diagnostics, Mannheim, Germany) diluted in DPBS for 1 h and incubated overnight in a rabbit anti-Tam antibody (TRITC polyclonal antibody, Invitrogen) diluted 1:200 in DPBS. After five times of 5 min wash in DPBS, immunostaining with a secondary goat anti-rabbit antibody, labelled with Alexa Fluor 633 (Thermo Fisher) diluted by 1:500 in DPBS, was carried out for 1 h. The tissues were washed again five times for 5 min in DPBS and stained with 4′,6-diamidino-2-phenylindole (DAPI, SERVA Electrophoresis) for 5 min. After a final wash with H_2_O, the sections were mounted in fluorescence mounting medium (Dako, Carpinteria, CA, USA).

Whole-brain images were acquired by scanning the prepared slides on a fluorescence imaging system AxioScan (Carl Zeiss) and by using confocal microscopy (Olympus FV1000, Olympus Corporation, Tokyo, Japan). Specific signals were quantified in two representative areas within the olfactory bulb, hypothalamus and cortex under standardized ×20 magnification using the software Netscope (Net-Base GmbH, Freiburg, Germany). Empty channel was used to exclude unspecific background signals, while specific signals were counted from appearance in Tam and Alexa Fluor 633 channel. Data graphing was performed using GraphPad Prism (version 9.0, GraphPad Software, USA).

### Statistical analysis

All statistical analyses were conducted using GraphPad Prism (GraphPad Software). Data are presented as mean ± standard deviation (SD). For comparisons between groups, one-way analysis of variance (ANOVA) was applied, followed by Tukey’s *post hoc* test for multiple comparisons. A *P*-value of <0.05 was considered statistically significant.

## Results

### Fluorescently labelled analogues of setmelanotide with disulphide replacement

The first set of peptides was based on the MC4R agonist setmelanotide (peptide **1.1**; Table 1). SPPS of setmelanotide backbone including acetylation of the *N*-terminus was carried out on TG R resin, while formation of the disulphide was achieved in solution. In contrast, lactamization in peptides **1.2–1.4** was carried out on resin. Therefore, the two cysteine building blocks were replaced with Fmoc-Glu(OPP)-OH and Fmoc-Dap(Mtt)-OH [diaminopropionic acid (Dap); methyltrityl (Mtt); phenylisopropyl ester (OPP)] in positions 2 and 8, respectively, to allow selective deprotection of the side chains. While peptide **1.2** was acetylated like setmelanotide, peptides **1.3** and **1.4** were fluorescently labelled by coupling Tam to the *N*-terminus, in case of peptide **1.4** with an aminohexanoic acid (Ahx) spacer. Analytical data of the purified peptides are summarized in the supporting information (Supplementary Table 1).

**Table 1:** Activity of setmelanotide variants in cAMP reporter gene assay

	Peptide^a^	EC_50_ (nM)	pEC_50_ ± SEM	*E*_max_ ± SEM (%)
**1**.**1**	Ac-R^b^CaHfRW^b^C-NH_2_	0.2	9.78 ± 0.07	100 ± 4
**1**.**2**	Ac-R^b^EaHfRW-^b^Dap-NH_2_	0.2	9.65 ± 0.12	112 ± 7
**1**.**3**	Tam-R^b^EaHfRW-^b^Dap-NH_2_	0.3	9.54 ± 0.14	131 ± 10
**1**.**4**	Tam-Ahx-R^b^EaHfRW-^b^Dap-NH_2_	0.7	9.19 ± 0.10	134 ± 7

Ac, acetylation; Ahx, aminohexanoic acid; Dap, diaminopropionic acid; EC_50_, half maximal efficient concentration; *E*_max_, maximal efficiency; SEM, standard error of the mean; Tam, 6-carboxytetramethylrhodamine.

^a^Peptides were tested on HEK293 cells transfected with the MC4R. *n* ≥ 2.

^b^Underlined amino acids indicate cyclization by disulphide or lactam for C-C and E-Dap, respectively.

Activity of setmelanotide and the variants was studied by a cAMP reporter gene assay. All peptides induced an increase of the luminescence signals in a concentration-dependent manner (Supplementary Fig. 1), and potencies are summarized in Table 1. Peptide **1.2** with lactam replacement of the disulphide showed equipotent activity as setmelanotide (peptide **1.1**) with EC_50_ = 0.2 nM and *E*_max_ of 112% ± 7% and 100% ± 4%, respectively. The *N*-terminal Tam labelling was well tolerated regarding receptor activity, as EC_50_ of peptide **1.3** was determined with 0.3 nM and the *E*_max_ value was slightly elevated with 131% ± 10%. Increasing distance of Tam label to the active pharmacophore in peptide **1.4** was the least tolerated modification, with slightly reduced EC_50_ = 0.7 nM and similar *E*_max_ as the other Tam-labelled setmelanotide analogue **1.3**.

### GhrR inverse agonists to reduce GhrR activity

GhrR inverse agonists were designed based on the lead structure KbFwLL-NH_2_ (peptide **2.1**). Synthesis was carried out on TG R resin with Fmoc-protected building blocks, and analytics of purified peptides are summarized in Supplementary Table 1. For Tam labelling in peptides **2.2–2.4**, Leu^6^ was replaced by Fmoc-Lys(Mmt)-OH and Boc-Lys(Boc)-OH [*tert*-butyloxycarbonyl (Boc); monomethoxytrityl (Mmt)] was used in position 1. After Mmt deprotection, Tam was coupled either directly (peptide **2.2**) or after four (peptide **2.3**) or six (peptide **2.4**) cycles of altering Fmoc-Sar-OH [sarcosine (Sar)] coupling and Fmoc deprotection (Table 2).

**Table 2:** Activity of GhrR inverse agonists on IP_1_ accumulation

	Peptide	EC_50_ (nM)^a^	pEC_50_ ± SEM^a^	*E*_min_ ± SEM (%)^a^
**2**.**1**	KbFwLL-NH_2_	69	7.16 ± 0.03	99 ± 2
**2**.**2**	Tam | KbFwLK-NH_2_	52	7.28 ± 0.51	10 ± 2
**2**.**3**	Tam-Sar_4_ | KbFwLK-NH_2_	104	6.98 ± 0.27	32 ± 4
**2**.**4**	Tam-Sar_6_ | KbFwLK-NH_2_	106	6.98 ± 0.22	28 ± 3

b, β-(3-benzothienyl)-D-alanine; Dap, diaminopropionic acid; EC_50_, half minimal efficient concentration; *E*_min_, minimal efficiency; Sar, sarcosine; SEM, standard error of the mean; Tam, 6-carboxytetramethylrhodamine.

^a^Stably transfected COS-7_GHSR1α cells were used for this assay. *n* ≥ 2.

All inverse agonists reduced the constitutive activity of the GhrR in a concentration-dependent manner, but efficiency varied significantly (Supplementary Fig. 2; Table 2). Peptide **2.1** was used as reference with EC_50_ = 69 nM and *E*_min_ = 99 ± 2. The compound with direct Tam labelling in the side chain of Lys^6^ (peptide **2.2**) maintained activity with EC_50_ = 52 nM but lost ∼90% of the minimal efficiency. Both variants with a Sar linker (peptides **2.3** and **2.4**) displayed a slight loss in activity with EC_50_ > 100 nM but regained 3-fold efficiency compared to peptide **2.2**.

### GhrR agonists as potential positive modulators of food intake

The third group of peptides in this study consisted of analogues of native GhrR agonist Ghr (Table 3). Ghr-based peptides were synthesized on a preloaded R-Wang resin from standard Fmoc-amino acid building blocks and Boc-Gly-OH in the *N*-terminal position. For synthesis of octanoylated Ser^3^ (peptide **3.1**), Fmoc-Ser(Trt)-OH was incorporated in the backbone, which allows selective Ser deprotection and coupling of octanoic acid (Oct) through an ester bond. For all other Ghr-based peptides, Ser^3^ was replaced by Dap. The Oct in peptides **3.2** and **3.5** was incorporated by coupling of the Fmoc-Dap(Oct)-OH building block in position 3, while for attachment of 3-phenylpropanoic acid (3PP, peptide **3.3**) and lauric acid (Lau, peptide **3.4**), the Fmoc-Dap(Mtt)-OH building block was used. Similarly, for palmitoylation of Lys^20^ (peptide **3.5**), Fmoc-Lys(Mmt)-OH was coupled in position 20 and palmitic acid (Pam) was attached following Mmt deprotection. For fluorescent labelling of peptides **3.2–3.5**, Fmoc-Lys(Dde)-OH [1-(4,4-dimethyl-2,6-dioxocyclohex-1-ylidene)ethyl (Dde)] was used in backbone position 16. This allowed for orthogonal deprotection of the Lys^16^ side chain, followed by Tam coupling. Purity analytics are listed in Supplementary Table 1.

**Table 3:** Activity of Ghr analogues

	Peptide	EC_50_ (nM)^a^	pEC_50_ ± SEM^a^	E_max_ ± SEM (%)^a^
**3**.**1**	GSS(Oct)FLSPEHQRVQQRKESKKPPAKLQRPR	1.7	8.77 ± 0.06	100 ± 2
**3**.**2**	GS-Dap(Oct)-FLSPEHQRVQQRK(Tam)ESKKPPAKLQRPR	1.6	8.81 ± 0.06	100 ± 2
**3**.**3**	GS-Dap(3PP)-FLSPEHQRVQQRK(Tam)ESKKPPAKLQRPR	2.2	8.65 ± 0.12	97 ± 5
**3**.**4**	GS-Dap(Lau)-FLSPEHQRVQQRK(Tam)ESKKPPAKLQRPR	3.2	8.49 ± 0.19	102 ± 8
**3**.**5**	GS-Dap(Oct)-FLSPEHQRVQQRK(Tam)ESKK(Pam)PPAKLQRPR	8.4	8.40 ± 0.09	101 ± 4

3PP, 3-phenylpropanoic acid; Dap, diaminopropionic acid; EC_50_, half maximal efficient concentration; *E*_max_, maximal efficiency; Lau, lauric acid; Oct, octanoic acid; Pam, palmitic acid; SEM, standard error of the mean; Tam, 6-carboxytetramethylrhodamine.

^a^IP_1_ accumulation was measured in stably transfected COS-7_GHSR1α cells. *n* ≥ 2.

Receptor activity of the GhrR agonist was measured in the same setup as inverse agonist activity but with reduced stimulation time (Supplementary Fig. 3). Peptide **3.1** and the Tam-labelled variant with Ser^3^Dap replacement (peptide **3.2**) showed highly comparable receptor activation with overlapping concentration–response curves (Supplementary Fig. 3) and similar EC_50_ values of 1.7 and 1.6 nM, respectively (Table 3). Also, the variation of the hydrophobic side-chain attachment in position 3 was well tolerated regarding receptor activation, as EC_50_ of peptides **3.3** and **3.4** were in the same range as Ghr. Peptide **3.5** with additional Pam on Lys^20^ showed a slight loss of activity with EC_50_ = 8.4 nM. All tested variants fully activated the receptor with efficiencies ranging from 97 to 102%.

### Permeability and stability of fluorescently labelled peptides evaluated on Calu-3 cells

The fluorescently labelled peptides were next tested in a cell-based permeability assay on Calu-3 cells (Supplementary Fig. 4). As reference, Fl (*P*_app_ = 112 · 10^−9^ cm/s ± 7 · 10^−9^ cm/s) and FD4 (*P*_app_ = 40 · 10^−9^ cm/s ± 4 · 10^−9^ cm/s) were included. Permeability values of Ghr analogues without additional palmitoylation (compounds **3.2**, **3.3** and **3.4**) were within the same range as FD4. In contrast, [Dap^3^(Oct),K^16^(Tam),K^20^(Pam)]Ghr (peptide **3.5**) was strongly less permeable with *P*_app_ = 5 · 10^−9^ cm/s ± 1 · 10^−9^ cm/s. Also, the inverse agonist **2.4** exhibits a low permeability value of <10 · 10^−9^ cm/s, while *P*_app_ for peptides **2.2** and **2.3** could not be determined as these peptides precipitated. The Tam-labelled setmelanotide variant **1.3** crossed Calu-3 cells with *P*_app_ = 16 · 10^−9^ cm/s ± 4 · 10^−9^ cm/s, and the setmelanotide variant with the additional Ahx (peptide **1.4**) linker showed similar permeability as the GhrR inverse agonist.

Besides permeability, degradation of all tested peptides during the permeability assay was monitored (Table 4). All short peptides (compounds **1.3**, **1.4** and **2.4**) showed high stability with intact compound ≥ 95% compared to room temperature reference. [Dap^3^(Oct),K^16^(Tam),K^20^(Pam)]Ghr was partly degraded with 78% intact compound remaining. In contrast, >50% of the three other GhrR agonists (peptides **3.2–3.4**) were degraded in the Calu-3 cell supernatant.

**Table 4:** Permeability and stability of compounds within 24 h on differentiated Calu-3 cells

	Peptide	*P*_app_ ± SEM (10^−9^ cm/s)	Intact peptide
**Fl**	Fluorescein.	112 ± 7	
**FD4**	4 kDa FITC-Dextran.	40 ± 4	
**1**.**3**	Tam-R^a^EaHfRW-^a^Dap-NH_2_.	16 ± 4	100%
**1**.**4**	Tam-Ahx-R^a^EaHfRW-^a^Dap-NH_2_.	9 ± 3	95%
**2**.**2**	KbFwLK(Tam)-NH_2_	Precipitation	
**2**.**3**	KbFwLK(Sar_4_-Tam)-NH_2_	Precipitation	
**2**.**4**	KbFwLK(Sar_6_-Tam)-NH_2_	9 ± 2	100%
**3**.**2**	[Dap^3^(Oct),K^16^(Tam)]Ghr	47 ± 13	37%
**3**.**3**	[Dap^3^(3PP),K^16^(Tam)]Ghr	41 ± 15	28%
**3**.**4**	[Dap^3^(Lau),K^16^(Tam)]Ghr	44 ± 13	12%
**3**.**5**	[Dap^3^(Oct),K^16^(Tam),K^20^(Pam)]Ghr	5 ± 1	78%

3PP, 3-phenylpropanoic acid; Ahx, aminohexanoic acid; b, β-(3-benzothienyl)-D-alanine; FITC, fluorescein isothiocyanate; Dap, diaminopropionic acid; Ghr, ghrelin; Lau, lauric acid; Oct, octanoic acid; Pam, palmitic acid; Sar, sarcosine; SEM, standard error of the mean; Tam, 6-carboxytetramethylrhodamine.

^a^ *n* ≥ 2. Underlined amino acids indicate cyclization by lactam.

### Uptake of fluorescently labelled peptides *in vivo*

Following *in vitro* characterization, the most suitable peptides were selected for *in vivo* experiments. Peptide **1.3**, from the setmelanotide group, was chosen due to its superior activity as well as permeability. Likewise, peptide **2.4** from the GhrR inverse agonist group was selected for its best activity and solubility. Furthermore, in the GhrR agonist group, peptide **3.2** demonstrated the best combination of activity, permeability and stability. In addition, peptide **3.5** was included to test whether the significantly lower permeability also transfers in the *in vivo* uptake. The selected peptides were administered intranasally to mice for varying time periods, ranging from 15 min to 24 h. Corresponding unlabelled peptides were used as negative controls. Later, the brain sections were evaluated by fluorescence imaging focusing on the olfactory bulb, the cortex and the hypothalamus.

To differentiate between the Tam-labelled peptides from unspecific fluorescence signals, additional analysis using an empty (green fluorescent protein) channel and immunohistochemical staining with an anti-Tam primary antibody, paired with a secondary antibody labelled with Alexa Fluor 633, was conducted. Signals that also appeared in the empty channel were classified as background fluorescence and excluded from quantification. Signals were considered specific only if they were concurrently detected in the anti-Tam channel.

**Figure 2:**
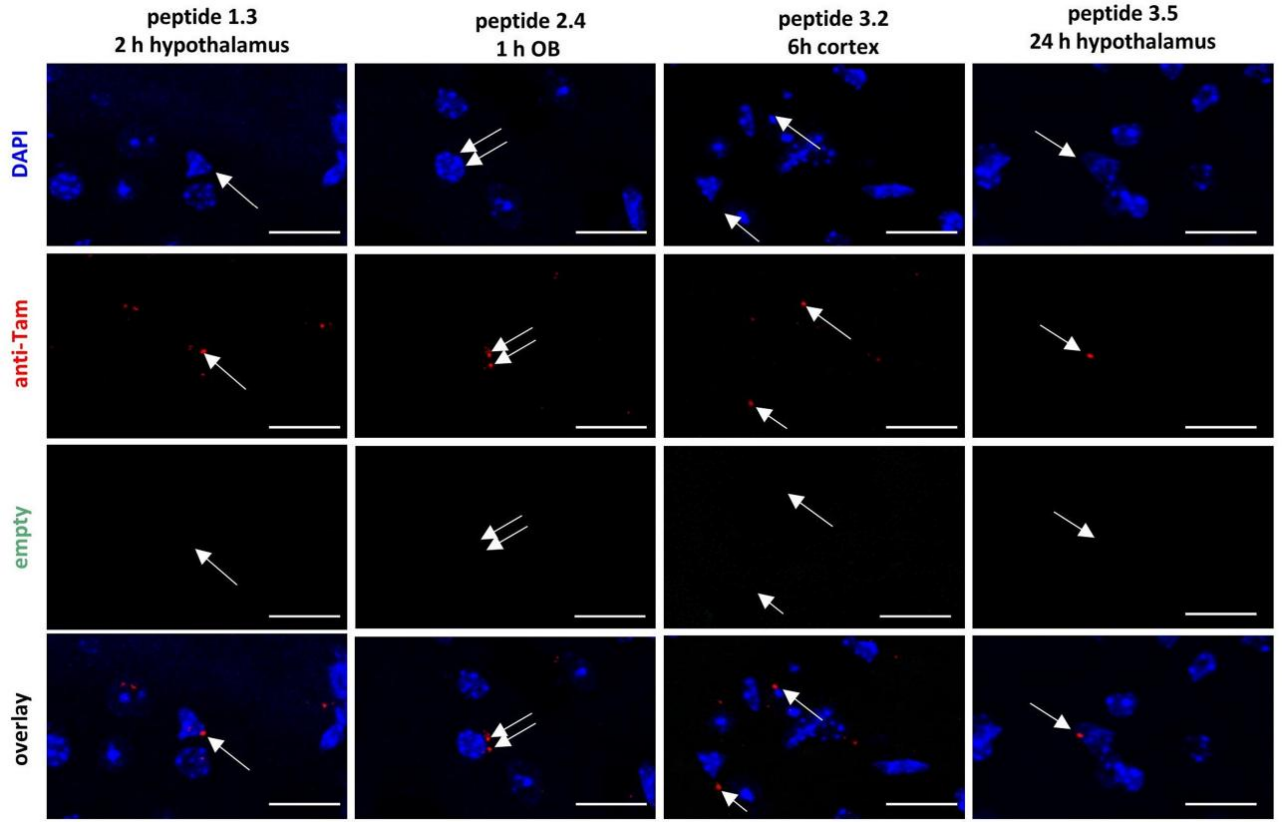
**Confocal microscopy for the detection of Tam-labelled peptides *in vivo*.** Representative images from mouse brains following intranasal administration at different time points and different brain regions. *n* = 3. Nuclei were stained with DAPI, Tam-labeled peptides were detected using anti-Tam antibody, and a separate channel was used to determine background fluorescence. Scale bar = 10 µm. DAPI, 4′,6-diamidino-2-phenylindole; OB, olfactory bulb; Tam, 6-carboxytetramethylrhodamine.

**Figure 3:**
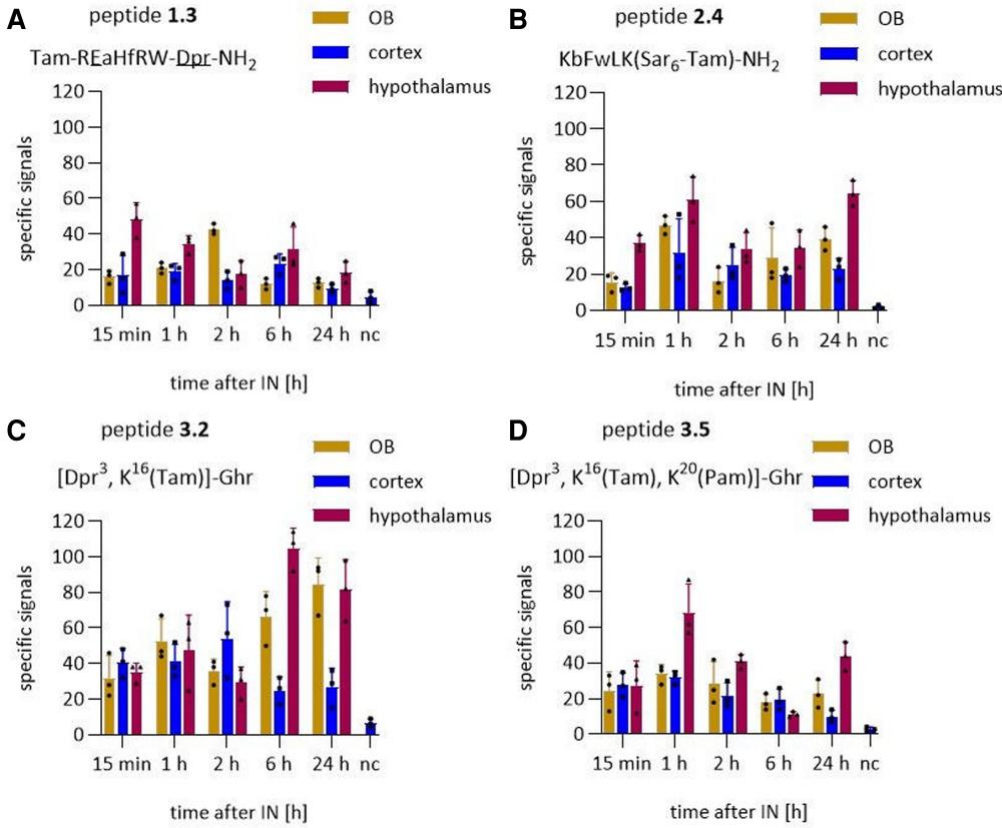
**Uptake of fluorescently labelled peptides after intranasal application.** Quantification of Tam-specific signals from fluorescent microscopy of the olfactory bulb, cortex and hypothalamus for peptide **1.3** (**A**), peptide **2.4** (**B**), peptide **3.2** (**C**) and peptide **3.5** (**D**) over time. Data are shown as mean ± SD. *n* = 3. Each data point represents one mouse. No statistical test was performed. b, β-(3-benzothienyl)-D-alanine; Dpr, diaminopropionic acid; Ghr, ghrelin; IN, intranasal application; nc, negative control. OB, olfactory bulb; Pam, palmitic acid; Sar, sarcosine; SD, standard deviation; Tam, 6-carboxytetramethylrhodamine.

In confocal microscopy (Fig. 2), specific signals localized near the cell nuclei were examined for all tested peptides in all three brain regions at the tested time points. Further, whole-slide scanning was performed. While these images showed increased background signals (Supplementary Fig. 5), signals in standardized areas of the olfactory bulb, the hypothalamus and the cortical area between them could be quantified (Fig. 3). For all peptides, signal counts were clearly over the negative controls for all examined regions and time points.

In the olfactory bulb, peptide **1.3** exhibited low but consistent signal counts across all time points but with a peak at 2 h. In the cortex, however, peptide **1.3** displayed more consistency. Furthermore, in the hypothalamus, peptide **1.3** exhibited the highest signal count among all regions, peaking at 15 min and 6 h. For peptide **2.4**, in the olfactory bulb, variations in signal count were observed, with the highest count at 1 h. In the cortex, the numbers of specific signals remained mostly stable. In addition, peptide **2.4** exhibited a high signal count overall in the hypothalamus, peaking at 1 and 24 h. Peptide **3.2** achieved the highest number of signals among the tested peptides. Within the first 2 h, the number of signals remained stable in the three brain regions. From 6 h on, the signal counts showed an increase for the olfactory bulb and the hypothalamus and a decrease in the cortex. Lastly, peptide **3.5** showed moderate signal counts in the olfactory bulb, with the highest recording at 1 h and another peak at 24 h. In the cortex and olfactory bulb, the signal count displayed a more stable pattern, with a slight decline from 6 h.

### Effect of intranasal peptides on food intake

**Figure 4:**
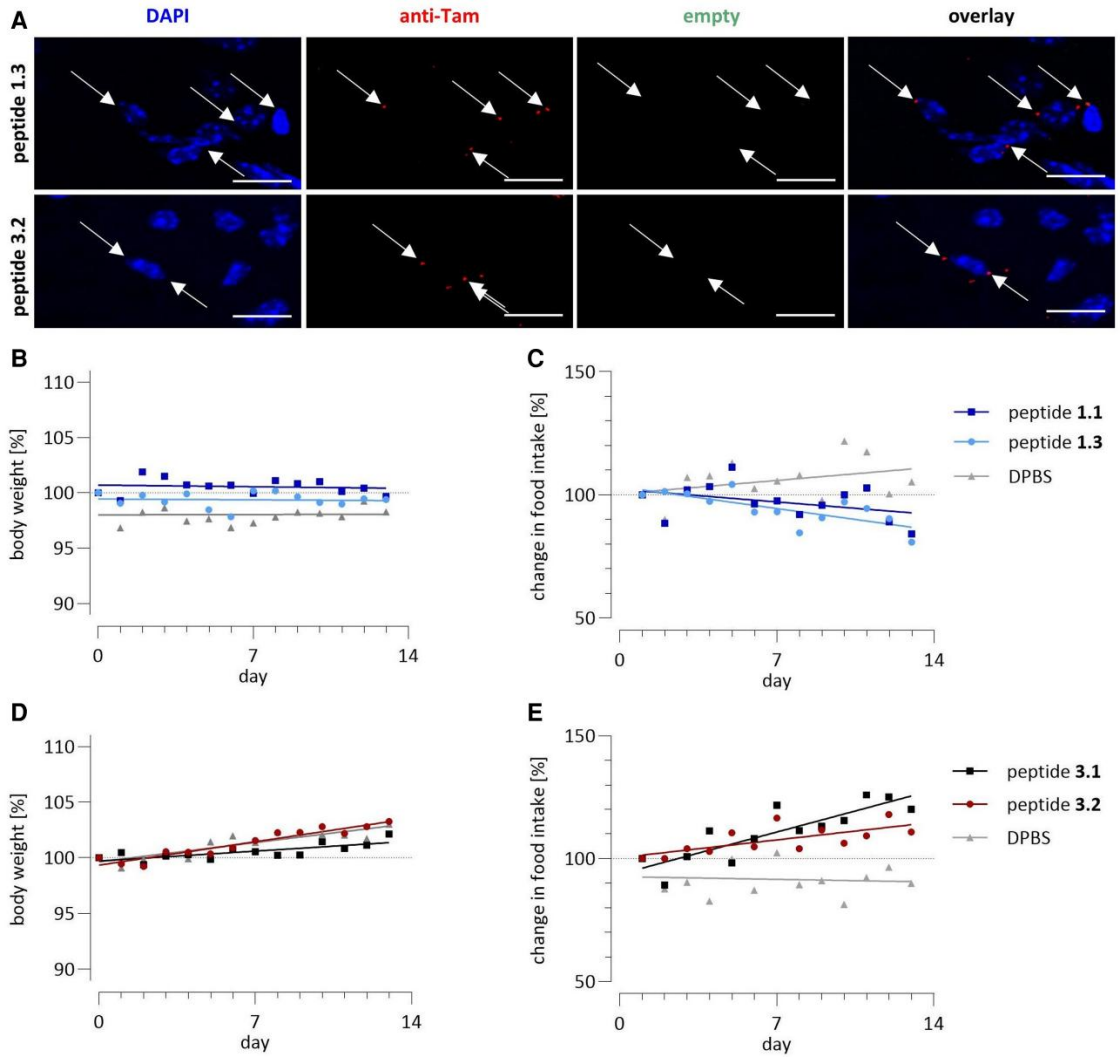
**Effect of intranasal peptides over 2 weeks.** Setmelanotide (**1.1**) (**B**, **C**) and Ghr (**3.1**) (**D**, **E**) and the fluorescently labelled analogues **1.3** and **3.2** were administrated intranasally once daily in male C57BL/6N mice, and relative changes in body weight (**B**, **D**) and food intake (**C**, **E**) were monitored. Data are displayed as mean. *n* ≥ 4. For control groups (analogues without fluorescent labelling and DPBS, *n* = 4), individual data points of each mouse are provided in Supplementary Fig. 6. Statistical analysis was performed using ANOVA followed by Tukey’s *post hoc* test; no significant differences were detected (*P* > 0.05). Confocal images (**A**) after daily intranasal peptides **1.3** and **3.2** from the hypothalamus. Microscopic images are representatives. Scale bar = 10 µm. DPBS, Dulbecco’s phosphate-buffered saline; IN, intranasal administration; Tam, 6-carboxytetramethylrhodamine.

As the administered peptides reached the hypothalamus, their effects on food intake and weight changes were analysed. Both unlabelled and fluorescently labelled peptides were administered intranasally over a 14-day period. The peptides were selected based on the number of specific signals they demonstrated, with one orexigenic and one anorectic peptide chosen. Peptide **3.2** was included due to its highest signals in the *in vivo* uptake studies, while peptide **1.3** was preferred over peptide **2.4** due to its superior activity and permeability. Throughout the 14-day intranasal administration of the peptides, daily body weight of the mice and percentile changes in food intake were monitored (Fig. 4). After intranasal administration over 14 days, the brains were examined by confocal microscopy (Fig. 4A). Vesicular-like specific signals have been detected in close proximity to the cell nuclei.

Mice treated daily with peptides **1.1** and **1.3** exhibited a tendency for reduced food intake compared to the control group. The body weight of all mice from the first group remained stable, independent of treatment. Meanwhile, daily administration of peptide **3.2** led to an ∼10% increase in the daily change in food intake. Conversely, the peptide **3.1** group exhibited a 20% change of increase, demonstrating higher food intake than the control group. All mice gained weight over the study period, with increases of 2–3.3%. Statistical analysis with ANOVA followed by Tukey’s *post hoc* test revealed no significant differences in the daily percentage change in food intake among treatment groups (*P* > 0.05), likely due to high inter-individual variability and limited sample sizes.

## Discussion

Peptides are involved in the regulation of food intake and show therapeutic potential for obesity and cachexia. Applying these compounds by the nasal route is a helpful tool to deliver the peptides to the hypothalamus and overcome major challenges of peptide therapeutics. Therefore, this work combined the use of peptide therapeutics with nose-to-brain delivery for the modulation of food intake focusing on the MC4R and GhrR as targets.

Within the hypothalamus, the GhrR and MC4R are two G protein-coupled receptors (GPCRs) involved in the regulation of energy homeostasis. Further, their activity can be modulated by peptide ligands. The orexigenic effect of the natural GhrR ligand Ghr was demonstrated by increased food intake in mice with intraperitoneal Ghr injection.^13^ In addition, the rise in preprandial Ghr levels induces the initiation of meals in humans.^12^ Interestingly, the GhrR shows a high constitutive activity, which was linked to food intake-promoting effects even in the absence of agonists.^15,16^ This basal activity and thereby food intake can be reduced with GhrR inverse agonists like KbFwLL-NH_2_.^20^ Another compound known for reduction of food intake is the MC4R agonist setmelanotide. The cyclic octapeptide was shown to successfully reduce food intake and body weight and was approved in 2020 for treatment of defined deficits within the leptin–melanocortin signalling pathway.^39,40^

Analogues of setmelanotide, KbFwLL-NH_2_ and Ghr were designed with the fluorescent label Tam. While Tam allows for tracking both in cell-based assays and in tissue, it can potentially affect properties like the interaction with the targeted receptor or peptide stability. Therefore, receptor activity was tested for all peptide analogues. First, the disulphide bond of setmelanotide, a known motive causing physiological degradation, was replaced by a lactam without loss in MC4R activity (peptide **1.2**).^41^ The compound with direct *N*-terminal Tam labelling (peptide **1.3**) showed a higher activity at the MC4R than peptide **1.4** with an Ahx spacer between the fluoro- and pharmacophore. For the Ghr inverse agonist, KbFwLL-NH_2_ (peptide **2.1**), Tam labelling in the side chain of Lys^6^ was selected, as lipidation in this position was well tolerated in previous studies.^35^ The Tam-labelled compound **2.2** maintained similar EC_50_ values as the lead compound but showed a clear loss of efficiency. This was partly restored by introducing a Sar linker between the peptide backbone and the fluorophore (peptides **2.3** and **2.4**), while the two different lengths of the linker tested did not differ in receptor activity. While octanoylation (or other hydrophobic moieties) in the side chain of Ser^3^ is a critical motive for Ghr to achieve GhrR activation, replacement by Dap^3^ stabilizes this bond in equipotent compounds.^42^ Also, Tam labelling in the side chain of Lys^16^ and palmitoylation in Lys^20^, a common motive to stabilize peptides by serum albumin binding, is well tolerated regarding GhrR activation.^35,43^

Besides receptor activation, permeability of compounds on differentiated Calu-3 cells, a model system for the nasal mucosa, was assessed. Thereby, two model compounds were included: Fl reflects permeability of a small molecule, while FD4 has a similar molecular weight as the Ghr analogues. All Ghr analogues except of the palmitoylated one (**3.5**) showed permeability comparable to FD4. As the peptides **3.2–3.4** were less stable in the assay setup, degradation products might add to the increased measured permeability. Rapid degradation of peptides by peptidases can generally occur in cell supernatants, particularly in the Calu-3 supernatant. The degradation pattern and dynamics of peptide **3.2** were previously published by our group,^33^ and structurally stabilized compounds are needed to allow full interpretation of permeability values. In contrast, the smaller peptides (**1.3**, **1.4** and **2.4**) show a detectable but low permeability while not being degraded. To assess whether these permeability rates correlate to the uptake *in vivo*, four peptides were selected for single nasal application in mice: the setmelanotide analogue **1.3** with best permeability and activity, the Ghr inverse agonist **2.4** meeting solubility criteria and the Ghr analogue **3.2** with best activity and permeability. In addition, peptide **3.5** was included to monitor whether low permeability in Calu-3 assay transfers to low *in vivo* uptake, which comparing the quantification of specific signals to peptide **3.2** holds true for these two peptides.

Nose-to-brain delivery of peptides can occur along different pathways.^44^ The neuronal pathways through the olfactory and trigeminal nerves create a direct connection between the nasal cavity and the brain.^45^ Thereby, compounds can be taken up intracellularly for transport along the neurons, which may take hours up to a day for drugs to reach the brain.^46^ In contrast, extraneuronal transport by the nasal route can enable drug delivery to the brain within minutes, but this mechanism requires the peptides to cross the nasal epithelium either trans- or paracellularly.^47,48^ Alternatively, drugs can enter the bloodstream from the nasal cavity and subsequently access the CNS by counter-current transfer.^49,50^ Based on these possibilities, mouse brains after nasal administration of labelled peptides were examined across five different time intervals, ranging from 15 min to 24 h, with focus on the olfactory bulb, the hypothalamus and the cortical region between them. For all evaluated peptides, vesicular-like structures were detected within the three regions. This vesicular-like accumulation of peptides is most likely the result of receptor-mediated internalization after stimulation with an agonist, as this is a common process for GPCRs including the Ghr and the melanocortin system.^51,52^ The variability of these signal counts highlights for contribution of different transport pathways at different time points.^53^ Peptide **1.3** exhibited rapid hypothalamic uptake with subsequent reduction, suggesting dominant extraneuronal transport. In contrast, for the Ghr-modulating peptides, signals increased within the hypothalamus at later time points, which might indicate additional intraneuronal transport.

As all peptides reached the hypothalamus, peptide **1.3** with a potential food intake-reducing effect and peptide **3.2** with a food intake-promoting effect were selected based on the best receptor activation. These peptides and the unlabelled setmelanotide **1.1** and Ghr **3.2** were administrated intranasally to mice once daily for 14 days, and the impact on food intake was monitored. Compared to the corresponding control group, mice with intranasal application of peptide **3.2** showed a positive trend on daily change in food intake, while mice treated with peptide **1.3** showed a negative trend on daily change in food intake. In addition, these trends are consistent within the group treated with the corresponding unlabelled compound.

In our *in vivo* experiments, selected peptides were administered intranasally once daily for 14 days to assess their effects on food intake. Although fluorescent labelling confirmed brain uptake and a trend of altered food intake was observed, these effects did not lead to significant changes in body weight under the current dosing conditions. Importantly, we acknowledge that this initial study did not include mechanistic analyses such as neuronal activation mapping (e.g. c-Fos staining) or the identification of specific hypothalamic targets (e.g. PVN and ARC). As a result of these limitations, it is not yet possible to fully define the central mechanisms underlying the peptides’ effects. Nonetheless, the observed brain uptake and changes in food intake provide a solid foundation for future studies involving extended dosing, higher concentrations and functional analyses to verify neuronal engagement and long-term physiological effects. While our study focused on intranasal administration to assess direct nose-to-brain delivery, future investigations comparing this route with systemic administration would provide further insights into the therapeutic potential. Furthermore, the detection of labelled peptides in cortical areas after 14 days of treatment shows that peptide diffusion is not limited to the hypothalamus. This raises the potential for off-target effects under chronic exposure. Future studies should explore targeted delivery strategies to improve regional specificity.

Previous studies with neuromedin-U receptor 2 agonists showed effective suppression of weight gain in mice when applied intranasally at 100 and 250 µg/day but not for 25 µg/day.^31^ As here <25 µg/day was applied, an increased dosage has a high potential to improve outcomes on body weight. Other approaches focus on the formulation, including the support of Ghr nose-to-brain delivery by liposomes.^54,55^ Such carrier systems can support especially the initial compound uptake. However, the detection of specific signals in the hypothalamus for all tested peptides indicates that formulations are not essential to allow peptide uptake from the nasal cavity. This is also in line with a report from Fehm *et al*.^56^ in 2001 on the fat-reducing effect of truncated melanocortin applied intranasally in humans, where peptides were also simply dissolved in water.

Taken together, the success of intranasal delivery opens new possibilities for non-invasive treatments of cachexia and obesity. Peptide-based treatments may provide a novel solution in clinical settings, especially for individuals resistant to existing weight management therapies or those experiencing adverse effects. In general, nose-to-brain delivery seems to be a suitable administration method for a high variety of peptides targeting receptors expressed in the hypothalamus.

## Conclusion

Peptides are highly involved in the regulation of food intake within the hypothalamus. Based on these processes, different peptides were identified to reduce or promote food intake. As demonstrated here for modulators of the melanocortin and Ghr system, nasal application allows successful delivery of these peptides to the hypothalamus. In addition, the observed effects on food intake underline the potential for future pharmaceutical treatment of obesity and cachexia by intranasally applied peptides. Including also previous reports, nose-to-brain delivery seems to be a method applicable for a broad range of peptides. However, the variation in the dynamics of the specific signals clearly indicates the involvement of different transport mechanisms and that their involvement most likely varies from compound to compound.

## Supplementary Material

fcaf450_Supplementary_Data

## Data Availability

The authors confirm that the data supporting the findings of this study are available within the article and its Supplementary material.
