# Supplementary material for: Modulating food intake by nasal application of peptides targeting melanocortin 4 receptor and ghrelin receptor systems
Source: Brain Commun. 2026 Jan 21;8(1):fcaf450. doi: 10.1093/braincomms/fcaf450 (PMC12820429; doi:10.1093/braincomms/fcaf450)
Supplement: fcaf450_Supplementary_Data [file fcaf450_supplementary_data.pdf]

Supplementary: Modulation Food Intake by Nasal Application of Peptides Targeting MC4R and GhrR Systems

Benginur Özbay [1†], Eva-Maria Jülke [2†], Moritz List[2], Marcin Nowicki[1], Sylvia Els-Heindl[2], Kerstin Immig[1], Karin Mörl[2], Ingo Bechmann[1]*, Annette G. Beck-Sickinger[2]*

[†]Benginur Özbay and Eva-Maria Jülke contributed equally to this work.
*Ingo Bechmann and Annette G. Beck-Sickinger contributed equally to this work.

Author affiliations:

1 Institute of Anatomy, Faculty of Medicine, University of Leipzig, 04103 Leipzig, Germany

2 Institute of Biochemistry, Faculty of Life Sciences, University of Leipzig, 04103 Leipzig, Germany

Correspondence to: Prof. Dr. Ingo Bechmann

Institute of Anatomy, Faculty of Medicine, Leipzig University, Liebigstraße 13, 04103 Leipzig, Germany
ingo.bechmann@medizin.uni-leipzig.de

Supplementary Information: Peptide Synthesis

Automated synthesis was carried out by a SYROI synthesis robot (MultiSynTech GmbH, Witten, Germany) on NovaSyn TG R resin (Merck KGaA, Darmstadt, Germany) or preloaded R-Wang resin (Iris Biotech, Marktredwitz, Germany) in a 15 µmol scale. Therefore, Fmoc-deprotection was performed with 40% piperidine (*v/v*, Sigma-Aldrich, St. Louis, Missouri, USA) in DMF for 3 min and subsequently with 20% piperidine (*v/v*) in N,N-dimethylformamide (DMF, VWR, Radnor, USA) for 10 min. Next, 8 eq. amino acid building block (Iris Biotech) was coupled with 8 eq. ethyl cyanohydroxyiminoacetate (Oxyma, Iris

Biotech) and 8 eq. N,N-diisopropylcarbodiimide (DIC, Iris Biotech) in DMF for two-times 42 min.

Manuel formation of peptide bonds was performed with 2-5 eq. amino or fatty acid (Sigma-Aldrich), and equal amount of hydroxybenzotriazole (HOBt, Merck) and DIC in 300 µL DMF for at least 6 h. In case of fatty acid coupling, an additional 100 µL of dichloromethane (DCM, VWR) was added to the reaction mix. For coupling of fatty acids through ester linkage, 5 eq. fatty acid, 5 eq. DIC, 5eq. HOBt, 5 eq. methylymidazole (MeI, Sigma-Aldrich) and dimethylaminopyridine (DMAP, Sigma-Aldrich) in 300 µL DMF were added to the resin and incubated overnight. Manuel Fmoc-deprotection was performed by incubating resins twice in 20% piperidine (v/v) in DMF for 10 min. Phenylisopropyl ester (OPP), trityl (Trt) from serin side chain, monomethoxytrity (Mmt) and methyltrityl (Mtt)-deprotection was achieved by 15-times 1 min incubation in 2% trifluoroacetic acid (v/v, TFA, Sigma-Aldrich) and 5% triisopropyl silane (v/v, TIS, Merck) in DCM and for 1-(4,4-dimethyl-2,6-dioxocyclohex-1-ylidene)ethyl (Dde) deprotection, resins were incubated 12-times in 2% hydrazine (Sigma-Aldrich) in DMF for 10 min. For lactamization, resins were incubated with 30 eq. HOBt and 30 eq. DIC in 300 µL DMF overnight at 40°C. Coupling of the fluorophore 6-carboxytetramethylrhodamine (Tam, ChemPep Inc., Wellington, USA) was carried out as double coupling each with 2 eq. Tam, 1.9 eq. HATU and 2 eq. DIPEA in 300 µl DMF for at least 3 h.

Full cleavage was performed with 7% thioanisole (TA, Sigma Aldrich), 3% ethane-1,2-dithiol (EDT, Sigma Aldrich) in TFA for 3 h. and peptides were precipitated and washed in ice cold diethyl ether. For formation of disulfide bond, peptides were dissolved in 5 mL 10 % acetonitrile (ACN, VWR) in water. 5 mL tris-buffered saline (TBS: 130 mM NaCl [Sigma-Aldrich], 25 mM tris[hydroxymethyl]aminomethane [tris, Carl Roth, Karlsruhe, Germany], 3 mM KCl [Grüssing, Filsum, Germany] was added and pH 7.6 was adjusted with HCl [Grüssing]), before incubation of the reaction mix under shaking for three days.

Supplementary Table 1: Analytics of synthesized peptides.

	peptide	M_{mono} [Da]	M_{exp} ($[M+H]^+$)	t_R[a] [% eluent B]	t_R [% eluent B]	purity
**1.1**	Ac-RCaHfRWC-NH_2	1116.6	1117.5	21.3	21.8[c]	≥ 95%
**1.2**	Ac-REaHfRW-Dap-NH_2	1109.6	1110.6	24.2	24.7[c]	≥ 95%
**1.3**	Tam-REaHfRW-Dap-NH_2	1479.7	1480.7	30.3	29.0[b]	≥ 95%
**1.4**	Tam-Ahx-REaHfRW-Dap-NH_2	1592.8	1593.8	40.9	34.5[c]	≥ 95%
**2.1**	KbFwLL-NH_2	907.5	908.5	57.8	46.9[b]	≥ 95%
**2.2**	KbFwLK(Tam)-NH_2	1334.6	1335.7	50.8	47.1[c]	≥ 95%
**2.3**	KbFwLK(Sar_4-Tam)-NH_2	1618.8	1619.8	50.3	40.5[b]	≥ 95%
**2.4**	KbFwLK(Sar_6-Tam)-NH_2	1760.9	1761.9	49.8	40.1[b]	≥ 95%
**3.1**	Ghr	3368.9	3369.9	33.3	30.1[b]	≥ 95%
**3.2**	[Dap^3(Oct),K^{16}(Tam)]Ghr	3780.0	3781.1	40.5	31.9[b]	≥ 95%
**3.3**	[Dap^3(3PP),K^{16}(Tam)]Ghr	3786.0	3787.0	38.8	31.0[b]	≥ 95%
**3.4**	[Dap^3(Lau),K^{16}(Tam)]Ghr	3836.1	3837.1	48.0	40.3[b]	≥ 95%
**3.5**	[Dap^3(Oct),K^{16}(Tam),K^{20}(Pam)]Ghr	4018.3	4019.3	52.3	44.8[b]	≥ 95%

Peptides were characterized by experimental mass (M_{exp}) corresponding to the calculated monoisotopic mass (M_{mono}). Purity of ≥ 95% was confirmed using reversed phase-high performance liquid chromatography on (a) Jupiter Proteo 4 µm 90 Å LC column (250 x 4.6 mm, Phenomenex) and (b) Aeris Peptide 3.6 µm XB-C18 100 Å column (250 x 4.6 mm, Phenomenex), (c) Kinetex 5 µm Biphenyl 100 Å LC column (250 x 4.6 mm, Phenomenex) by a linear gradient of eluent B in eluent A increasing by 1%/min. Abbreviations: Ahx: 6-aminohexanoic acid; b: β-(3-benzothienyl)-D-alanine; Dap: diaminopropionic acid; Ghr: ghrelin. Lau: lauric acid; Oct: octanoic acid; Pam: palmitic acid; Tam: 6-carboxytetramethylrhodamine

Supplementary Fig. 1: Concentration-response curves of Tam-labeled setmelanotide analogues.

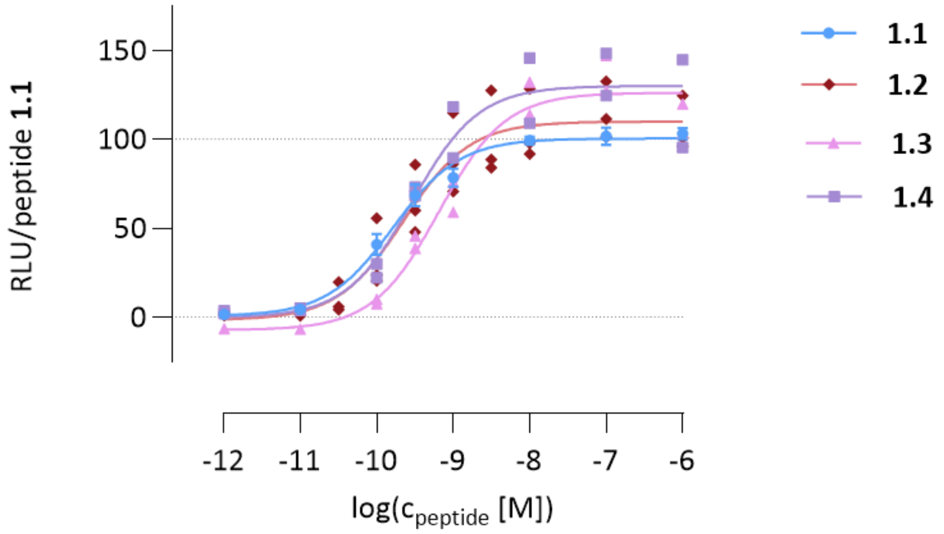

Activity was measured by a cAMP based reporter gene assay on HEK293 cells transfected with the melanocortin 4 receptor (MC4R). Data are shown as mean ± SEM for groups with n > 10. For groups with n < 10 individual data points are shown, each representing an independent experiment. No statistical test was performed. Abbreviations: cAMP: cyclic adenosine monophosphate; RLU: relative luminescence units; SEM: standard error of the mean.

Supplementary Fig. 2: Concentration dependend reduction of inositolmonophosphate accumulation mediated by ghrelin receptor inverse agonist.

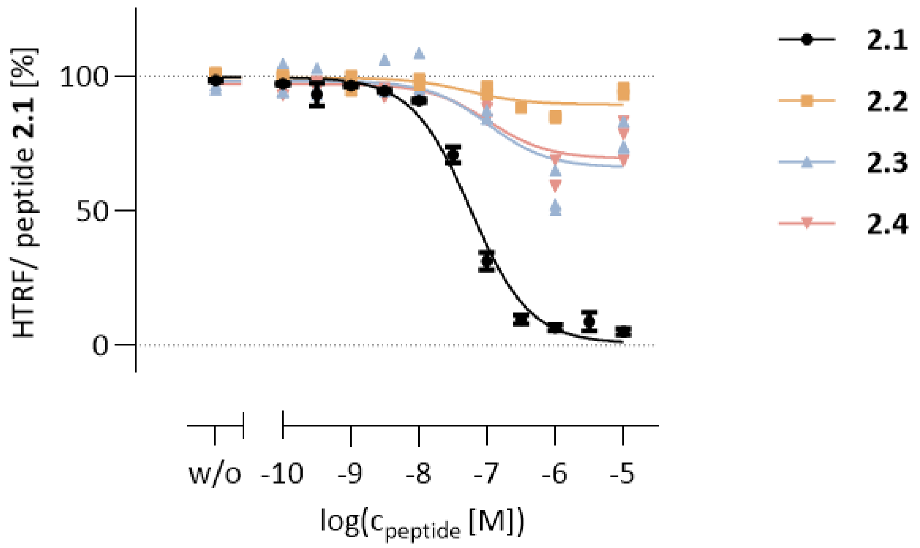

Concentration-response curves were measured in stably transfected COS-7_GHSR1α cells. Data are shown as mean ± SEM for groups with n > 10. For groups with n < 10 individual data points are shown, each representing an independent experiment. No statistical test was performed. Abbreviations: HTRF: homogeneous time-resolved fluorescence; SEM: standard error of the mean.

Supplementary Fig. 3: Concentration-response curves of ghrelin agonists.

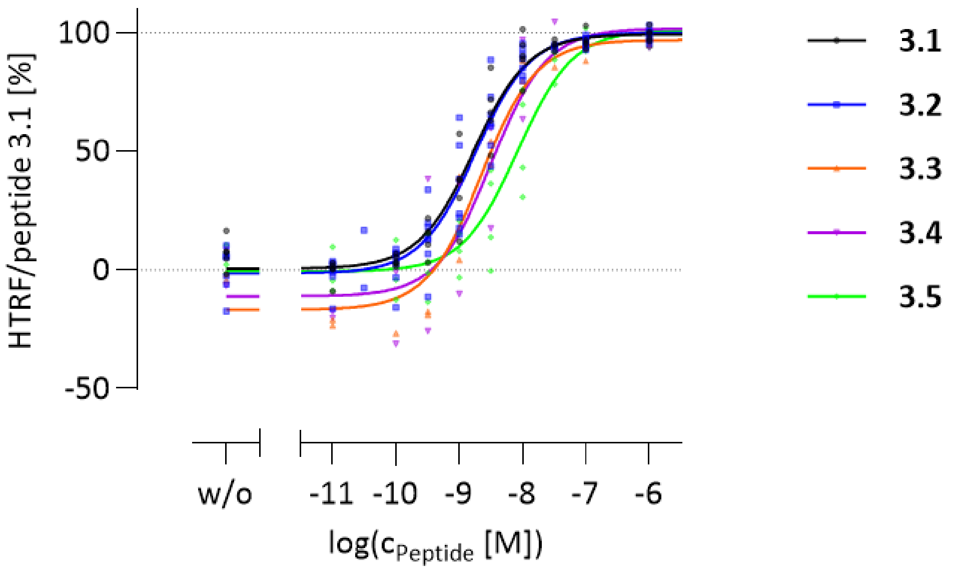

Stably transfected COS-7_GHSR1α were used to measure inositolmonophosphate accumulation. Data are shown as mean ± SEM for groups with n > 10. For groups with n < 10 individual data points are shown, each representing an independent experiment. No statistical test was performed. Abbreviations: HTRF: Homogeneous time-resolved fluorescence; SEM: standard error of the mean.

Supplementary Fig. 4: Apparent Permeability (P_{app}) of fluorescently labeled peptides through differentiated Calu-3 cell layer.

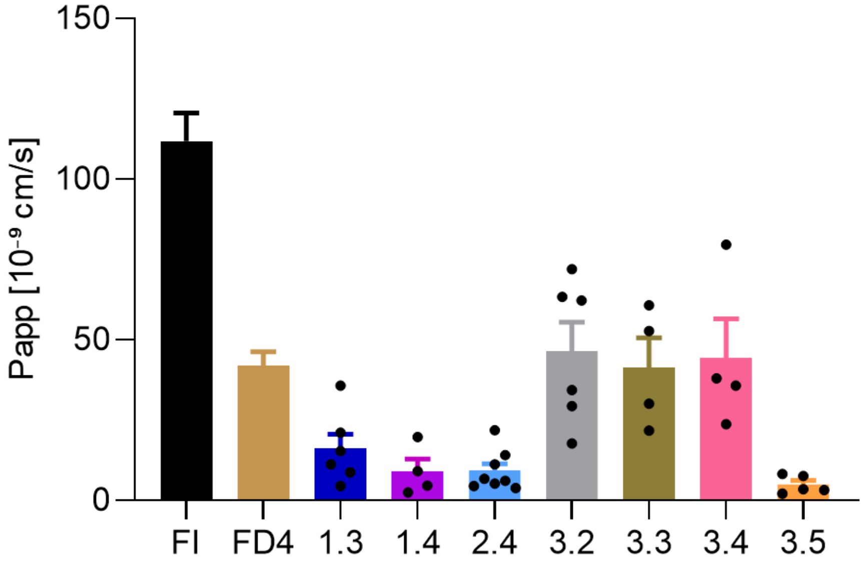

Data are shown as mean ± SEM for groups with n > 10. For groups with n < 10 individual data points are shown, each representing an independent experiment. No statistical test was performed. SEM: standard error of the mean.

Supplementary Fig. 5: Axioscan microscopy for the detection of Tam-labeled peptides *in vivo.*

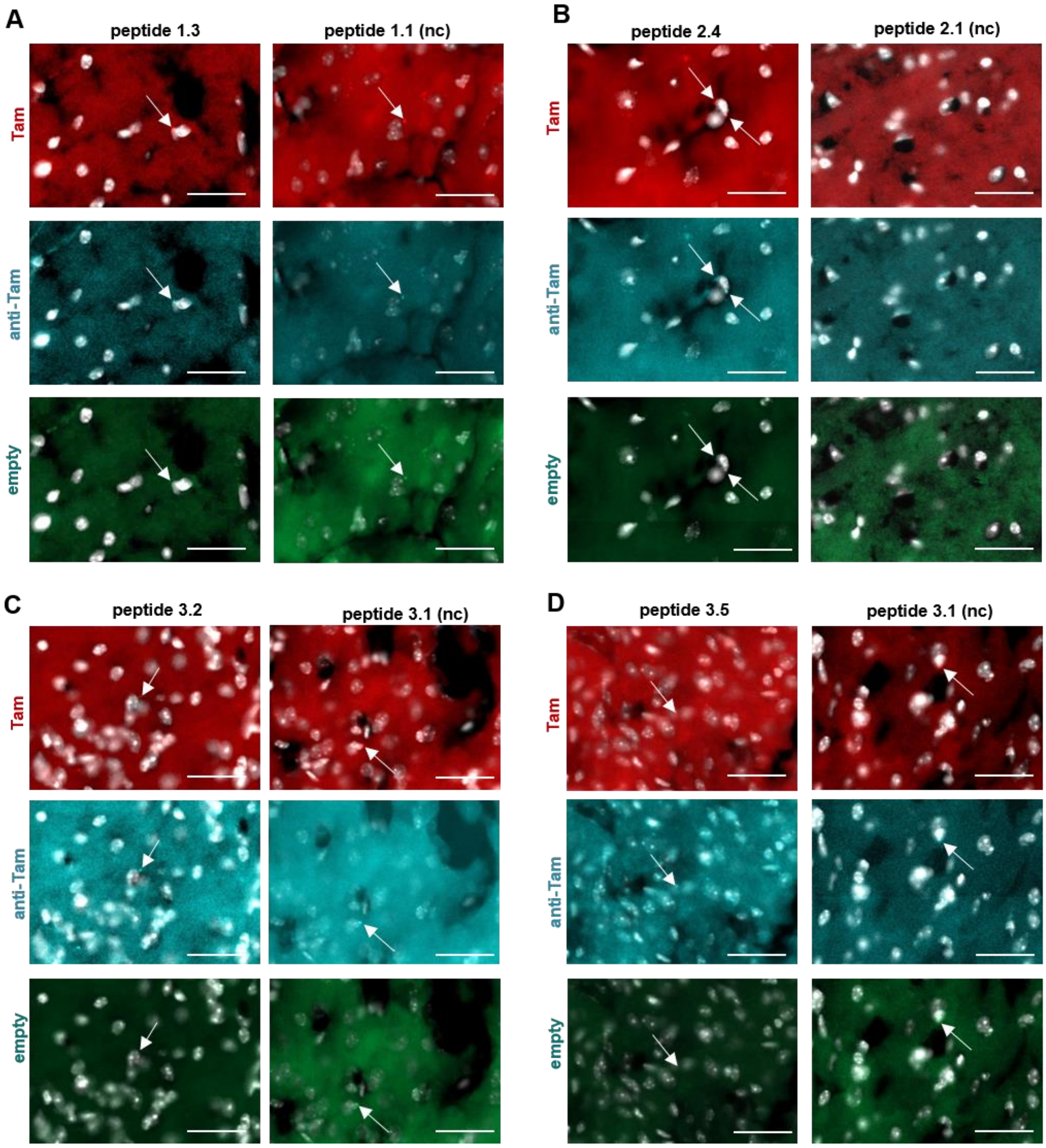

Axioscan microscopy images from hypothalamus 24 h after intranasal administration of fluorescence labeled peptide 1.3 (A), peptide 2.4 (B), peptide 3.2 (C), peptide 3.5 (D) with corresponding unmodified peptides. Images are representative of n = 3 (n: independent experiments). Nuclei from DAPI staining are shown in white, Tam fluorescence in red, anti-Tam antibody labeling in turquoise and empty channel for determination of background fluorescence in green. Scale bar = 50 µm. Abbreviation: DAPI: 4,6-diamidino-2-phenylindole, nc: negative control, Tam: 6-carboxytetramethylrhodamine.

Supplementary Fig. 6: Daily percentage changes in body weight and food intake

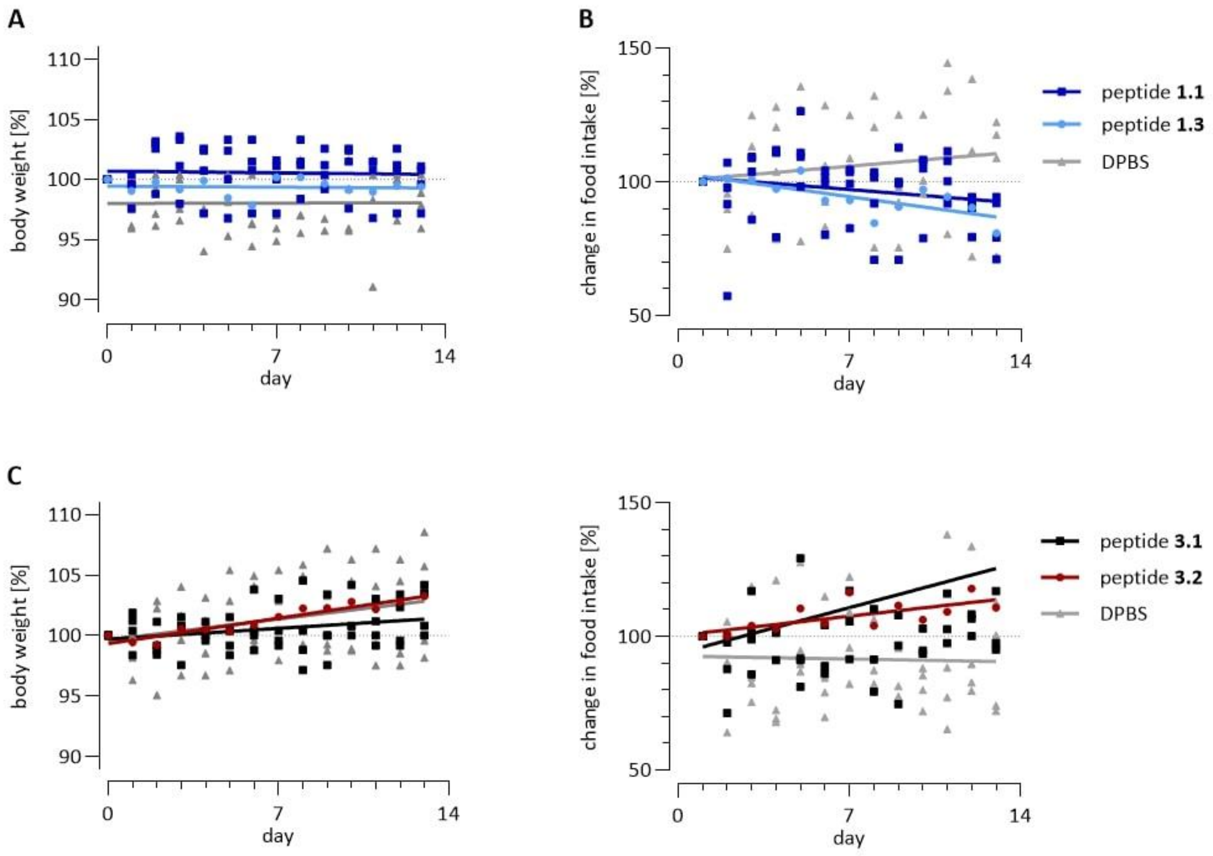

Setmelanotide (1.1, A, B) and ghrelin (3.1, C, D), as well as their fluorescently labeled analogues 1.3 and 3.2, were administered intranasally once daily to male C57BL/6N mice. Relative changes in body weight (A, C) and food intake (B, D) were monitored. Data are presented as mean for n = 10. For control groups (analogues without fluorescent label and DPBS, n = 4) individual data points are shown. This data respresents the same data as figure 4, with individual data points displayed. Each point represents one individual mouse. Abbreviations: DPBS: Dulbecco's phosphate-buffered saline.
